# Patient-Specific Age: The Other Side of the Coin in Advanced Mesenchymal Stem Cell Therapy

**DOI:** 10.3389/fphys.2015.00362

**Published:** 2015-12-02

**Authors:** Magdalena M. Schimke, Sabrina Marozin, Günter Lepperdinger

**Affiliations:** Department of Cell Biology and Physiology, Stem Cell Research, Aging and Regeneration, University SalzburgSalzburg, Austria

**Keywords:** vascular niche, cell-based therapy, aging biology, cellular dysfunction, age-associated pathology, regenerative medicine

## Abstract

Multipotential mesenchymal stromal cells (MSC) are present as a rare subpopulation within any type of stroma in the body of higher animals. Prominently, MSC have been recognized to reside in perivascular locations, supposedly maintaining blood vessel integrity. During tissue damage and injury, MSC/pericytes become activated, evade from their perivascular niche and are thus assumed to support wound healing and tissue regeneration. *In vitro* MSC exhibit demonstrated capabilities to differentiate into a wide variety of tissue cell types. Hence, many MSC-based therapeutic approaches have been performed to address bone, cartilage, or heart regeneration. Furthermore, prominent studies showed efficacy of *ex vivo* expanded MSC to countervail graft-vs.-host-disease. Therefore, additional fields of application are presently conceived, in which MSC-based therapies potentially unfold beneficial effects, such as amelioration of non-healing conditions after tendon or spinal cord injury, as well as neuropathies. Working along these lines, MSC-based scientific research has been forged ahead to prominently occupy the clinical stage. Aging is to a great deal stochastic by nature bringing forth changes in an individual fashion. Yet, is aging of stem cells or/and their corresponding niche considered a determining factor for outcome and success of clinical therapies?

## Background

Mesenchymal stem cells (MSC) are multipotential precursor cells that maintain and repair tissues in the body of adult individuals. MSC have been isolated from embryonic tissues as well as from adult up to advanced ages (Landgraf et al., 2011; Batsali et al., 2013; Beane et al., 2014a; Todeschi et al., 2015). Yet experimental knowledge about *in vivo* activities in regenerating models is still scarce (Wang et al., 2013a; Zhao et al., 2015). Besides replenishing mesenchymal tissues, MSC also modulate haematopoiesis as well as immune response (Pontikoglou et al., 2011; Hao et al., 2012; Law and Chaudhuri, 2013; Bianco, 2014). Conceivably residing in perivascular locations, MSC are identified with cells better known as pericytes. This cell type is involved in maintaining blood vessel integrity under normal conditions. During tissue damage and injury, MSC are thought to become instantaneously activated and by evading from their perivascular niche to support wound healing and tissue regeneration (Murray et al., 2014; Wong et al., 2015).

MSC are acknowledged for their potential to regenerate damaged tissue due to their ability to terminally differentiate into a broad variety of cell types. Deliberately, stem cells are perceived being ageless by nature. Yet, it is by now generally accepted that, with advancing age, a decline of stem cell function and activity has its share in delaying the replacement and the turnover of damaged cells in compromised renewable tissues (Bajek et al., 2012; Bethel et al., 2013). Also, stem cells in their niches are exposed to threads such as reactive oxygen species, harmful chemical agents or physical stresses, which trigger premature senescence, provoke accelerated cell death or cellular transformation (Li et al., 2014a). In osseous tissues at an advanced age, both mass and mineral density of cortical and cancellous bone steadily decreases. At the same time, fat cells emerge within the bone marrow and muscles. Fat cell-specific expedition of systemically deteriorating adipokines and pro-inflammatory cytokines primes the emergence of age-associated diseases. Hence, aged or senescent circumstances call for advanced therapies (Reitinger et al., 2015). Scientific approaches aiming at standardized medical treatment often neglect these biological and patho-physiological constraints. Nevertheless, these should be distinctly considered. Otherwise rightly conceived and diligently established strategies are bound to fail.

## Unresolved questions regarding phenotypic appearance and *in vitro* techniques

### Biological properties

Stromal cell types exhibit characteristic features. The rather large spindle-shaped cells present microvilli on their surface and produce extracellular matrix, which together facilitates MSC to firmly adhere to cell culture plastic (Friedenstein, 1976; Castro-Malaspina et al., 1980). This property is often exploited to isolate and culture-purify MSC from biopsies (Owen and Friedenstein, 1988). Variant culture conditions significantly impact on cell adhesion and consequently isolation outcome and MSC expansion. Therefore, inconsistencies often arise when employing inappropriate brands of cell culture plastic and media supplements.

### MSC immunophenotype

Another selection criterion for MSC is a tri-lineage differentiation potential forming osseous, adipose, and cartilaginous progenitors (Mark et al., 2013; Patrikoski et al., 2014), and a distinguished immune phenotype positive for CD105, CD73, and CD90, and negative for CD45, CD34, HLA-DR, and other markers (Dominici et al., 2006; Al-Nbaheen et al., 2013). This marker canon is not always unequivocal, as other cell types may also fulfill these criteria. MSC-like cells often exhibit differential marker expression depending on tissue origin and period of culture expansion (Gronthos et al., 1999; Wagner et al., 2005; Kaiser et al., 2007; Riekstina et al., 2008). A prominent example is the surface marker STRO-1. Due to the availability of a highly affine monoclonal antibody, STRO-1 has not only gained popularity as a marker but also for use in cell enrichment (Stewart et al., 1999). Endothelial cells may however also express STRO-1 thus questioning the specificity of this marker (Lin et al., 2011; Ning et al., 2011). Though, the likely equivalence of MSC to vascular pericytes reconciles STRO-1 being a good marker for true MSC (Feng et al., 2011; Chen et al., 2012; da Silva Meirelles et al., 2015). Another currently debated marker is CD34. Previously, MSC were considered CD34 negative, yet adipose-derived MSC express CD34 (Lin et al., 2008; Baer, 2014). Likewise, CD271 and CD146 markers have also been described (Rasini et al., 2013; Busser et al., 2015; Cuthbert et al., 2015). During culture expansion marker expression can change. Whether, these changes reflect the biological age of MSC has not been thoroughly studied. Also specific markers amenable for the quantification of MSC age are so far not available. In appreciation of this experience, further standards in MSC validation are needed, in particular when surface antigens expression differs in primary vs. culture-expanded MSC. Together with the restricting biological constraint of biological and replicative age, also tissue origin has to be accounted.

### Isolation techniques

To enhance isolation yields, often cell purification and fractionation by density gradient centrifugation is performed. This procedure bears the enhanced risk of contamination and therefore requests skilled operators. To reduce variability, closed, semi-automated separation devices, granting higher recovery rates, have been engineered. Their operational speed and efficiency warrant reproducible processing thereby, easing standardization for fulfilling compliance criteria in “Good Manufacturers Practice” (GMP) (Ito et al., 2010; Otsuru et al., 2013, 2015). Production of clinical-grade MSC must be performed in accordance to GMP standards and compels not only reproducibility, but also scalability. Working along the same line, novel automated cell platforms have been introduced for production of higher cell numbers in reduced times and passages (Roberts et al., 2012; Nold et al., 2013; Rojewski et al., 2013; Hanley et al., 2014). Gaining a pure MSC culture is difficult in particular when attempting to erase single-lineage committed progenitors or contaminating hematopoietic cells (Kerk et al., 1985; Kuznetsov et al., 1997). Therefore, selection enrichment using fluorescence-activated cell sorting (FACS) or magnetic-activated cell sorting (MACS) has been introduced. Although FACS was initially favored because it provides higher purities, shear stress within the fluids compromised cell viability, and chemotaxis (Deschaseaux et al., 2003; Rada et al., 2011; Li et al., 2013).

### Culture conditions

Further open issues are (i) seeding densities in a defined growth medium (Ben Azouna et al., 2012; Hagmann et al., 2013), (ii) specification of media supplements (Aldahmash et al., 2011; Bieback et al., 2012; Chimenti et al., 2014; Stern-Straeter et al., 2014), and (iii) atmospheric oxygen conditions during culture and handling (Grayson et al., 2006; Klepsch et al., 2013; Ito et al., 2015). Not solely in culture expansion, serum is also often used in cryopreservation. The use of animal-derived serum bears contamination risks and lot-to-lot variability. For clinical translation, it is therefore reasoned to replace animal-derived supplements (Tekkatte et al., 2011). Using autologous human serum has been proposed (Stute et al., 2004). Arguments such as high costs and the high likelihood of factors in the blood of donors, which may dominantly impact on MSC growth, greatly promoted the development of serum-free, chemically defined media (Mimura et al., 2011; Chase et al., 2012; Li et al., 2015). Alternatively, standardized human blood-derived products have been proposed (Díez et al., 2015; Riordan et al., 2015). Still unresolved and seemingly important in establishing MSC for therapeutic application is the definition of potency assays, that address patient's age.

## Moving into clinics

In recent years, more than 500 clinical trials employing MSC for the treatment of various diseases have been registered worldwide (http://www.clinicaltrials.gov, 60 thereof in Europe www.clinicaltrialsregister.eu). Besides applications in musculoskeletal defects and trauma MSC are also widely tested in pathologies, which are of immunological etiology such as graft-vs.-host disease or multiple sclerosis, lupus, diabetes type I, and Crone's disease. The reason is, MSC exhibit dominant immune-modulatory properties (Castro-Manrreza and Montesinos, 2015). More and more details regarding the underlying molecular mechanisms and cellular interactions how MSC control immune competent cells are being unraveled (Glenn and Whartenby, 2014). MSCs are also tested for liver and heart pathologies as well as ocular diseases (Li et al., 2014b).

### Clinical trial cohort variability

Many approaches are still exploratory and many ongoing clinical trials are still in Phase I Most strategies were carefully validated in diligently designed preclinical tests. However, commencing clinical trials firstly address safety issues thus rarely corroborating results cannot be expected. Early phase clinical studies also often comprise small heterogeneous groups exhibiting variant health status, age and ethnicity. Also sex-specific differences appear to be of relevance (Tajiri et al., 2014). For example, MSC from female bone marrow could be smaller and lower in number (Zanotti et al., 2014) but divided more rapidly than found for most male MSC. They had higher clonogenic activity and exhibited enhanced expression of the surface antigens, CD119 and CD130 (Siegel et al., 2013). Furthermore, functional differences were reported to be sexually dimorphic. Suppression of T-lymphocyte proliferation is more marked in females, while male-derived MSC possess a more robust osteogenic activity (Siegel et al., 2013; Ranganathan et al., 2014; Park et al., 2015). Female MSC showed a higher resistance in endotoxic and hypoxic injury models, inferring that improved survival in adverse micro-environments may be dependent on sex steroids. Indeed estrogen and estradiol exert a protective activity against apoptosis, favor proliferation, and delay the onset of senescence (Huang et al., 2013; Li et al., 2014c; Sung et al., 2015).

Most trials refrain from stratifying into age groups for obvious reasons of addressing a specific pathology rather than a gerontological principle. Animal trials are mostly done on young animals; clinical trials are rarely concerned with young or middle aged adults. MSC for animal experimentation are most often isolated from young animals, instead in humans autologous approaches are performed mostly in aged individuals. Further concerns are the greatly varying cell isolation and expansion procedures, as the outcome of a clinical trial is pertinently influenced by the quality of a cellular product. In fact, the population of cells harvested from bone marrow aspirates is very heterogeneous, consisting only of 0.001–0.01% mesenchymal precursor cells. To gain sufficient numbers of potential stem cells, the isolates are purified for subsequent *in vitro* expansion. At that point the uncertainty arises whether replicating cells accumulate damage which turns them senescent (Bonab et al., 2006). Suffice it to say that the recommendation is to restrict applications to replicating young MSC.

### MSC aging: Cellular changes and determinants

First animal studies showed, already 10 years ago, that the transplantation of aged rat MSC was less effective (Zhang et al., 2005). Hence, the vexed question arose whether donor age influences the therapeutic efficacy in clinical trials (Wang et al., 2013b). MSC derived from old patients exhibit reduced proliferative capacity and in some cases show skewed multi-lineage differentiation potentials, telomere shortening, or DNA damage accumulation (Behrens et al., 2014; Efimenko et al., 2014; Kizilay Mancini et al., 2015; Reitinger et al., 2015). They also exhibit increased levels of reactive oxygen species and nitric oxide, lower superoxydismutase activity senescence-associated β-galactosidase activity, enlarged morphology, and p53 protein upregulation. These observations confirm the doubts that aged cells may only be acceptable for transplantation if specially treated or selected (Kornicka et al., 2015). In cases of autologous cell-based applications, constitution, and health status of the patient may pertinently impact on the therapeutic outcome. Changes in MSC morphology, proliferation capacity, senescence, and multi-lineage potential are linked to advanced age but could be also induced by several disease conditions, thus compromising their therapeutic potencies (Sethe et al., 2006; Choudhery et al., 2014; Escacena et al., 2015).

**Figure 1:**
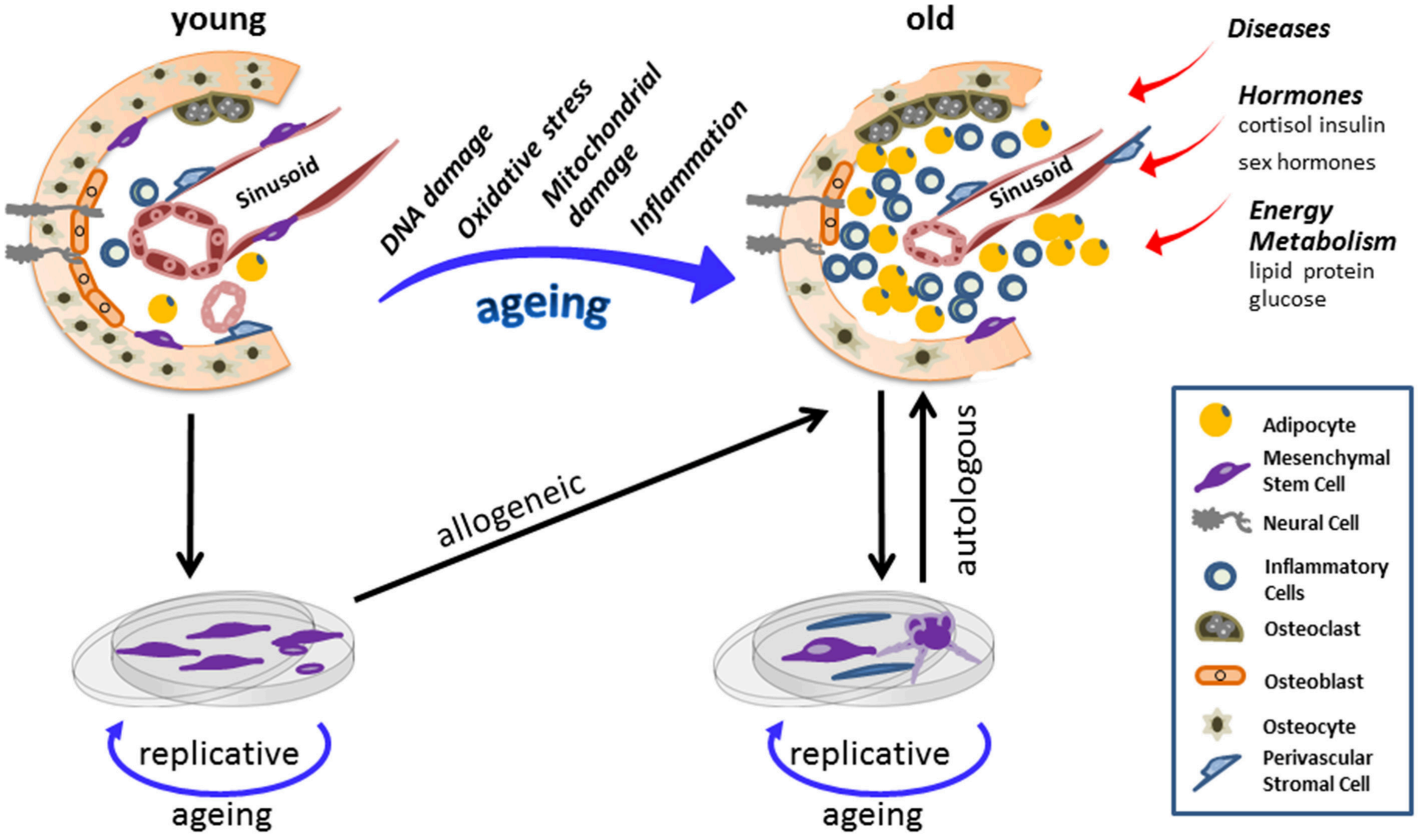
**Stem-cell populations are maintained in “niches.”** Stem cells and the corresponding niche entertain dynamic interactions controlling sustaining tissues regeneration and repair, yet also regulate somatic maintenance. Here a scheme of a bone marrow niche is provided and its potential changes as a consequence of the aging process. Chronological aging diminishes adult stem cells fitness and on the long run induces cellular and anatomical modifications within the niche. In old age mesenchymal stem cell (MSC) population has shrunken, while the number of inflammatory and fat cells increased to a large extent. Alongside, other extracellular factors also influence the aging process of stem cells together with their corresponding niche. Explanation of stem cells and *in vitro* amplifications adds risks of replicative aging to stem cell and progenitor fates. Hence when reintroducing cells in an autologous way, which is more likely undertaken as patients are more often of advanced age, chronologically old/replicative aged cells are applied. Conclusively, aberrations within a stem cell niche, as a consequence of aging, impose both chronological and replicative aging on therapeutic MSC, potentially compromising the success of autologous transplantation in aged donors.

It is aged people, who are the primary targets for stem cell therapy. Provided putative deviations, procedures selecting for flawless cells are potentially required before applying aged cells in autologous therapy. Allogenic transplantation of stem cells derived from young donors are considered to potentially overcome aging-related limitations (Figure 1).

It is further conceivable that the epigenetic status and/or aging as well as pathology-specific changes of cells may be different when isolating MSC from different tissues. It is currently unclear how to predict the optimal MSC source in order to warrant an optimal outcome (Golpanian et al., 2015). Interventions provoking rejuvenation of MSC have therefore been proposed. Extracellular factors, such as oxygen tension or redox status, exert effects on MSC aging (De Barros et al., 2013; Bigot et al., 2015; Sart et al., 2015).

Microenvironmental conditions, epigenetic processes including DNA methylation, telomerase activity, microRNA as well as specific growth factor signaling actively modulate MSC fate (Madonna et al., 2013; Jing et al., 2015; Oh et al., 2015; Okada et al., 2015). For their role in MSC biology, all these aspects provide challenging means to further enhance stem cell efficacy. Yet to date methods need to reliably prompt efficacy before being implemented in cell production at authorized manufacturing sites (Oh et al., 2014). Measuring the cellular fitness of MSC has been suggested being a invaluable prognostic value for enhancing the therapeutic success (Wagner et al., 2010). This complex question may however only be tackled by generating a comprehensive database for comparative purposes that stores information on source and MSC characteristics derived from different disease conditions. It is very likely that a pathological state perturbs the tissue milieu (niche) where MSC reside (Mastri et al., 2014). A systemically challenged micro-environment will impinge on the functional integrity of MSC, acknowledging the widely accepted principle of a stem cell niche (Krinner and Roeder, 2014). MSC niche interactions appear to be of mutual benefit since MSC release a wide range of bioactive factors that confer trophic and immune-modulatory effects, such as cytokines and distinct forms of miRNA and tRNA species (Hsiao et al., 2012; Baglio et al., 2015). More and more reports have unraveled the potent features of the MSC secretome, which now is thought to become by itself a regenerative therapeutical tool (Sdrimas and Kourembanas, 2014; Gallina et al., 2015; Succar et al., 2015; Tran and Damaser, 2015).

Considering the knowledge that the patient's disease state pertinently affects MSC functionality, the impact of medication on MSC, be it before or after implantation, is largely neglected and little data are available. MSC are greatly resistant to chemotherapy agents and therefore, therapies in patients undergoing cancer treatment are conceivable (Beane et al., 2014b; Bosco et al., 2015; Yoon et al., 2015) but have yet been not consistently successful (Buttiglieri et al., 2011; Choron et al., 2015). Medications, such as immune suppressants, glucocorticoids, psychopharmaceutics, and contrast agents may interfere with cell viability and proliferation capacity (Georgiou et al., 2012; Jansen Of Lorkeers et al., 2014; Schneider et al., 2015; Tang et al., 2015; Tsuji et al., 2015). These studies provided preliminary though valuable evidences, suggesting the need of more consistent drug interaction studies with special attention given to both short and long-term observations.

## Conclusion

MSC are considered “work horses” for cell-based therapy. This is because very little ethical concerns have been raised and it is now widely accepted that MSC are largely resistant to malignant transformation. Being ubiquitously present in many tissues, MSC could be successfully isolated from cord blood, fat, skeletal muscles, dental pulp, and several other sources (Ogura et al., 2014). Despite all positive aspects and the remarkable progress in stem cell research, many decisive issues remain to be resolved. Difficult standardization on one hand, the persisting ambiguity regarding MSC-specific markers and the technical challenges with orthotopic transplantations are hampering experimental validations. It is important to unambiguously prove that stromal tissues of various origins contain stromal stem progenitor cells. In contrast to bone marrow-derived MSC, most experimental procedures aiming at identifying MSC in different tissues were solely based on *in vitro* data regarding non-clonal culture and multi-lineage differentiation. Therefore, the true stem cell character of these MSC has yet not be fully proven (Bhartiya, 2013). For these reasons, their physiology and functions *in vivo* are still scarcely known. Currently, information regarding biological and molecular features, as well as the dominant influences on their surrounding tissue environment, appears insufficient (Murray et al., 2014; New et al., 2015). In fact, sharing the same immune phenotype may be a deceptive indicator for predicting cellular function. Further “known unknowns” are details about migratory properties of MSC. MSC are likely incapable of entering the circulation (Hoogduijn et al., 2014). However, short-distance migration into adjacent tissues is conceivable (Vanden Berg-Foels, 2014), implying that a population of MSC in any given tissue might indeed represent a mixture of local and “migrant” MSC.

In light of these considerations, the question whether special MSC sources are putatively more potent in specific disease treatments requires in depth studies, addressing comparative measures of therapeutic efficacy and safety. Several studies albeit designed and performed in a comparable fashion reported that superficially equal MSC resulted in different outcomes *in vivo* (Meraviglia et al., 2014; Wang et al., 2014; Reinisch et al., 2015). To overcome the present discrepancies, US Food and Drug Administration (FDA) suggested building a database, in which data from MSC subjected to varying culture conditions, derived from diverse tissues, and donors should be compiled.

Once safety of MSC administration can be granted, the next step is to obtain consistent results on sustained curative benefit. Inconsistency or failure in clinical outcomes might not be solely related to MSC preparations, but also attributable to disparate therapeutic protocols. The optimal route of MSC delivery and dosage regime are still debated (Kean et al., 2013; Richardson et al., 2013; Chang et al., 2014; Yavagal et al., 2014). The success of a systemic delivery depends very much on the ability of MSC to home to the site of injury and to access target tissues in case of a damaged or still regenerating vascularization (Cerri et al., 2015). Efficacious outcomes after intravenous delivery have repeatedly been reported (Semedo et al., 2009; Cruz et al., 2015; Rapp et al., 2015). Needless to say that topic administration could be more successful (Antunes et al., 2014; Ishihara et al., 2014; Cerri et al., 2015; Huang et al., 2015).

Specification and definition of efficacy is another pending question to be answered. Multi-lineage differentiation *in vitro* has often been employed to deliberately grant *in vivo* efficacy. As the one has very little to do with the other, in particular because differentiation *in vitro* is performed under tightly controlled culture conditions, better methods have been conceived. *In vivo*, MSC differentiation or complex functions certainly depend on a plethora of parameters, which can be hardly reconstructed *in vitro*. Direct interactions with micro-vascular structures as well as with other cell types, such as endothelial cells appear to play stimulating and inducing roles (Chen et al., 2015; da Silva Meirelles et al., 2015; Tsai et al., 2015). Working along this line, the ability of MSC to suppress T- and B-lymphocytes proliferation and to produce lymphokines was exploited to predict MSC efficacy *in vivo* (Collins et al., 2014; Del Fattore et al., 2015). But once more controversial results have been reported (Sajic et al., 2012).

This pointed at establishing superior methods to verify the functional capacity of MSC prior clinical applications. Thus, tests in living animal were proposed to distinctly assay intrinsic capacities to differentiate into functional tissues or to exert other desired functions. Sequential heterotopic transplantation has proven to fulfill this need. Generation of heterotopic ossicles has demonstrated not only skeletogenic potentials of single clone progenitors but also self-renewal ability. It must be stressed that *in vivo* functional assays also provided means and measures to simultaneously acquire long-term safety data. There is a lack of suitable long-lived models, which would allow the sensitive risk assessment of MSC or its secretome in inducing malignancy (Bruno et al., 2014; Arango-Rodriguez et al., 2015; Schweizer et al., 2015).

A major caveat in most clinical MSC applications is the lack of long-term follow ups. Such studies would reveal those parameters that are essential in determining risk and safety issues in cell therapy. Despite some recent attempts (Brizuela et al., 2014; Caminal et al., 2014; Ciccocioppo et al., 2015; Daltro et al., 2015; Davatchi et al., 2015), we are currently far from a systematic and robust analysis. Only little attention has been given to acquire information on survival after implantation, on the impact of MSC cell therapy on other tissues or on unintended alterations in target tissue as well as in the tissue-borne MSC population itself. Implementation of MSC in therapies raises many practical questions which are beyond scientific consideration. Presently most trials are on a small-scale and mainly performed by academic centers. To acquire reliable and precise information on how to efficiently introduce MSC in clinical therapy, larger trials are needed with the active involvement of industry and the support of interdisciplinary teams.

## Author contributions

All authors helped with drafting and revision of the article, GL completed the manuscript. All authors approved the submitted version.

### Conflict of interest statement

The authors declare that the research was conducted in the absence of any commercial or financial relationships that could be construed as a potential conflict of interest.
